# Positive Response to Thermobalancing Therapy Enabled by Therapeutic Device in Men with Non-Malignant Prostate Diseases: BPH and Chronic Prostatitis

**DOI:** 10.3390/diseases4020018

**Published:** 2016-04-18

**Authors:** Ivan Gerasimovich Aghajanyan, Simon Allen

**Affiliations:** 1 Department of Urology, Yerevan State Medical University, 9 Ezras Hasratyan St, Yerevan 0052, Armenia; aghajanyan_hov@yahoo.com; 2 Fine Treatment, 29 Rewley Road, Oxford OX1 2RA, UK

**Keywords:** chronic prostatitis, CP/CPPS, chronic pelvic pain, benign prostatic hyperplasia, BPH, prostate natural treatment, therapeutic device, thermobalancing therapy, prostatic disease

## Abstract

Background: The most common types of non-malignant prostate diseases are benign prostatic hyperplasia (BPH) and chronic prostatitis (CP). The aim of this study was to find out whether thermobalancing therapy with a physiotherapeutic device is effective for BPH and CP. Methods: During a 2.5-year period, 124 men with BPH over the age of 55 were investigated. Clinical parameters were tested twice: via the International Prostate Symptom Score (IPSS) and via ultrasound measurement of prostate volume (PV) and uroflowmetry maximum flow rate (Q_max_), before and after six months of therapy. In 45 men with CP under the age of 55, the dynamics of the National Institute of Health Chronic Prostatitis Symptom Index (NIH-CPSI) were studied. Results: The results of the investigated index tests in men with BPH confirmed a decrease in IPSS (*p* < 0.001), a reduction in PV (*p* < 0.001), an increase in Q_max_ (*p* < 0.001), and an improvement of quality of life (QoL) (*p* < 0.001). NIH-CPSI scores in men with CP indicated positive dynamics. Conclusions: The observed positive changes in IPSS, PV, and Q_max_ in men with BPH and the improvement in NIH-CPSI-QoL in patients with CP after using a physiotherapeutic device for six months as mono-therapy, support the view that thermobalancing therapy with the device can be recommended for these patients. Furthermore, the therapeutic device is free of side effects.

## 1. Introduction

The prevalence of histologically diagnosed prostatic hyperplasia increases from 8 percent in men aged 31–40, to 40–50 percent in men aged 51–60, and to over 80 percent in men older than age 80 [1]. Epidemiologic data suggest that the prevalence of chronic prostatitis (CP) is comparable to ischemic heart disease and diabetes mellitus, as about 8.2% men are believed to be affected [2]. Thus, benign prostate hyperplasia (BPH) and CP are common diseases in males, and BPH is often complicated by CP [3]. At the same time, chronic inflammation in the prostate gland plays an important role in the development of BPH [4]. Moreover, inflammation may be considered a key component of BPH pathogenesis, in addition to androgen receptor signaling in tissue remodeling typical of the advanced stages of the disease [5].

Accumulating evidence suggests that inflammation may contribute to the development of BPH and LUTS. The inflammatory injury may contribute to cytokine production via inflammatory cells driving local growth factor production and angiogenesis in the prostatic tissue. Acceptance of this suggestion can lead to novel treatment strategies [6]. The first-line treatments in CP have always been antibiotics and non-steroidal anti-inflammatory drugs (NSAIDs) [7]. A new potential target for medical therapy of LUTS due to BPH is CP and consequently prostatic inflammation. Drugs currently investigated for the treatment of this inflammation include the hexanic lipidosterolic extract of *Serenoa* *repens*, nonsteroidal anti-inflammatory drugs, and vitamin D receptor agonists [8].

Although there are some suggestions that NSAIDs increase the risk of prostatic diseases, most studies suggest that NSAIDs have the potential to improve symptoms in, and reduce the risk of, prostatic diseases [9]. The systematic review has shown that NSAIDs compared to a placebo, or in addition to known BPH therapy, improve BPH symptoms as measured using the IPSS and peak urinary flow rate, so authors believe that their study provides proof of the concept that inflammation in BPH can be a real target for medical research and targeted intervention [10].

However, non-selective NSAIDs should be used with caution, especially in older people, after other safer treatments have not provided sufficient pain relief. The lowest dose should be provided, and for the shortest duration. NSAIDs should be prescribed especially carefully to older people, with monitoring for gastrointestinal, renal and cardiovascular side effects, and drug–disease interactions [11].

At the same time, since 2009, when Simon Allen invented the non-invasive and safe thermobalancing therapy, enabled by the use of a therapeutic device for the treatment of chronic internal diseases, empirical data were obtained that the device he designed provides the positive and clinically meaningful effect in men with CP and benign prostatic hyperplasia [12]. A clinically controlled trial involving 124 men with BPH, to whom the therapeutic device was administered exclusively as a mono-therapy, has confirmed the effectiveness and safety of thermobalancing therapy [13]. A clinical study on 45 men with CP has also been completed. Thus, in the present study, we investigated the effect of the continuous use of the therapeutic device on patients with prostatic disease.

## 2. Materials and Methods

### 2.1. Study Design

The observational clinical controlled study was used. Enrollment began on April 2013 in the Department of Urology of the Yerevan State Medical University. The Ethics Committee of the Yerevan State Medical University approved the clinical study on thermobalancing therapy enabled by the use of a physiotherapeutic device. Effectiveness of the therapeutic device was studied by comparing men with BPH, who received treatment with the therapeutic device, with a control group in the watchful waiting stage, and by also comparing patients with CP who received treatment with the therapeutic device with the control no-treatment group. Dynamics of the symptoms and the indicators in each group were evaluated in comparison to their data in the beginning and end of the treatment, before and after a 6-month period of time.

### 2.2. Evaluation

The baseline evaluations included a complete physical examination, medical history, DRE, serum biochemistry, PSA measurements, electrolytes, and urine and renal function tests. Evaluations were made at baseline and 6 months after the treatment. International Prostate Symptom Score—Quality of Life (IPSS-QoL) scores were used in men with BPH, and the National Institute of Health Chronic Prostatitis Symptom Index (NIH-CPSI) scores were used in patients with CP. Prostate volume (PV) was measured at baseline and at 6 months after the treatment via ultrasonography (US-9000E2 ultrasound scanner, Rising Medical Equipment Co. Ltd., Beijing, China), and uroflowmetry (maximum urinary flow rate—Q_max_, mL/s) was used for the measurement of the rate of urine flow parameters (Sanuro2UL, Santron Meditronic, Maharashtra, India). The standard ellipsoid formula length × width × height × 0.52 was used to determine prostate volume.

### 2.3. Participants and Interventions

226 men were examined in April 2013, and 124 with BPH were selected for the clinical trial. Inclusion criteria: Men were eligible for enrolment if they were over the age of 55, in the absence of acute prostatitis, at the level of prostate-specific antigen (PSA) not more than 4 m·mol/L, and with a Qmax higher than 5 mL/s. It did not matter if they were treated at the time of enrollment with medicines. However, after the use of the therapeutic device, other treatments were cancelled gradually. Exclusion criteria: PV greater than 60 mL and co-morbidities, such as diabetes, heart failure, cancer, *etc.*

Thus, 80 men were excluded, as their PV was over 60 mL or they had severe co-morbidities; 10 preferred an operation; four were suspected of prostate cancer; and eight did not attend the following examinations. The quantity of patients in the treatment and control groups was similar (124 men).

**Figure 1:**
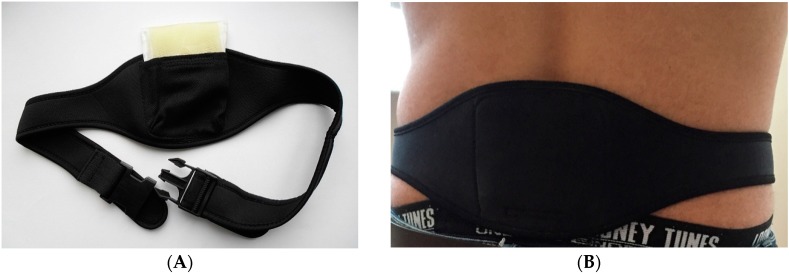
The therapeutic device alone (on the **A**) and the device tightly applied to the coccyx area in the projection of the prostate (on the **B**).

In this study, we also present part of another study that included the dynamic of quality of life (QoL) in 45 patients with chronic nonbacterial prostatitis (Cat III), also known as CP/CPPS, before and after the 6-month period of the use of the physiotherapeutic device, and in 45 men in the control group that did not use the device. Men in treatment groups were given therapeutic device, termed Allen’s Device (see Figure 1).

### 2.4. Statistical Analysis

Because the independent samples *t*-test and pair samples *t*-test is only suitable for interval and ratio data, the Wilcoxon signed-rank test by using SPSS was conducted.

## 3. Results

### 3.1. Urinary Symptoms and QoL

**Figure 2:**
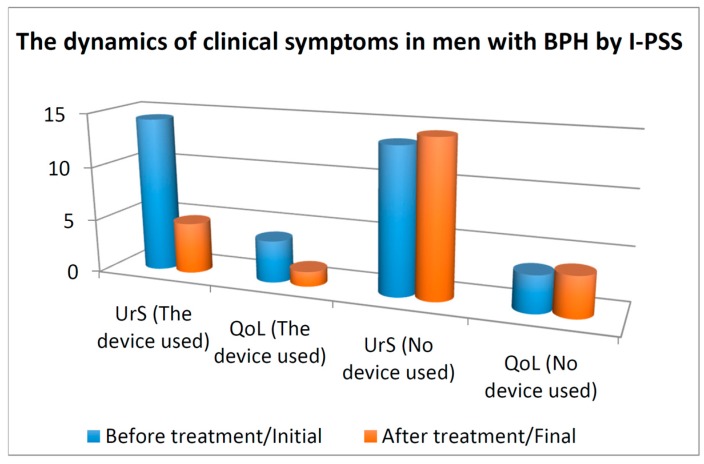
International Prostate Symptom Score (IPSS), urinary symptoms (UrS), and quality of life (QoL) in 124 patients with benign prostate hyperplasia (BPH) on thermobalancing therapy and in the control group, 124 men with BPH in watchful waiting at the beginning and at the end of the study.

In the control group, the mean IPSS-UrS increased from 13.45 ± 3.254 to 14.35 ± 3.396, whereas in the treatment group the mean IPSS-UrS decreased from 14.33 ± 3.399 to 4.73 ± 2.754 at the end of the observation period. For the control group, the z value was 6.018 with a *p* value (*p* < 0.001). For the treatment group, the z value was 9.674 with a significance level (*p* < 0.001). This indicates that the treatment with the therapeutic device decreased the urinary symptoms significantly, while in the absence of treatment the symptoms increased significantly, see Figure 2.

In the control group, the mean IPSS-QoL increased from 3.43 ± 0.956 to 3.76 ± 0.983, whereas in the treatment group the mean IPSS-QoL decreased from 3.91 ± 0.755 to 1.39 ± 1.110. For the control group, the z value was 5.286 with a *p* value (*p* < 0.001). For the treatment group, the z value was 9.672 with a *p* value (*p* < 0.001). These results indicate that the treatment with the therapeutic device improved the QoL, while in the control group the QoL worsened, see Figure 2.

### 3.2. Prostate Volume and Q_max_

In the control group the mean prostate volume increased from 45.54 to 50.85 mL, whereas in the treatment group the mean prostate volume decreased from 45.19 to 31.86 mL. For the control group, the z value is −8.727 at the significance level (*p* < 0.001). Thus, there was a statistically significant increase in the prostate volume in the control group. For the treatment group, the z value is −9.669 at the significance level (*p* < 0.001). Thus, the treatment with the therapeutic device reduced the PV level significantly, whereas in the no-treatment group the PV level increased.

**Figure 3:**
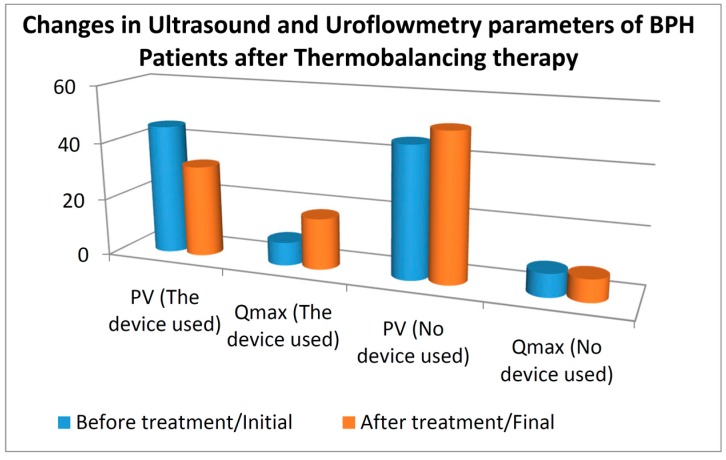
The changes in prostate volume (PV) mL and the uroflowmetry (maximum urinary flow rate (Q_max_, mL/s) in 124 patients with BPH on thermobalancing therapy and in the control group, 124 men with BPH in watchful waiting, at the beginning and at the end of the study.

In the control group, the mean Q_max_ decreased from 7.95 ± 2.871 to 7.7 ± 2.695 mL/s, whereas in the treatment group the mean Q_max_ increased from 8.10 ± 3.041 to 17.73 ± 4.392 mL/s. For the control group, the z value was 1.929 and the *p* value was 0.054 (>0.05), indicating no statistically significant difference. For the treatment group, the z value is 9.621 at the significance level (*p* < 0.001), indicating a significant increase in the Q_max_. Thus, our results demonstrate that the therapeutic device increased the uroflowmetry Q_max_ significantly in BHP patients, whereas the control group had no significant difference in the uroflowmetry Q_max_. See changes in PV and Q_max_ on Figure 3.

### 3.3. Quality of Life Score in Patients with Chronic Prostatitis

**Figure 4:**
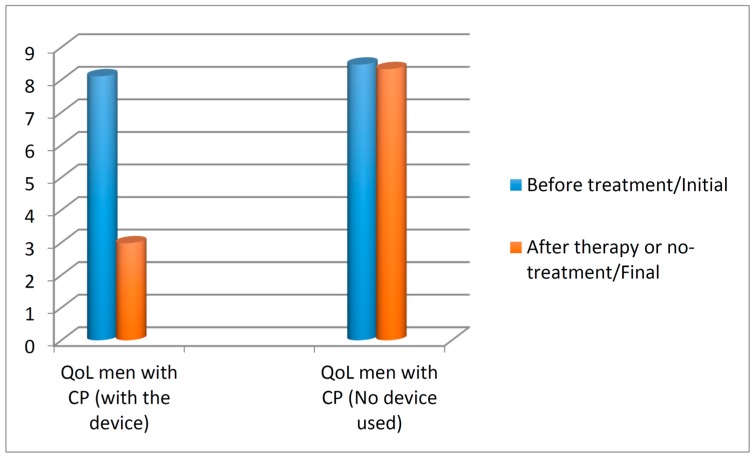
National Institute of Health Chronic Prostatitis Symptom Index (NIH-CPSI)—Quality of Life (QoL) score in 45 patients with chronic prostatitis/chronic pelvic pain syndrome (CP/CPPS) on thermobalancing therapy and in the control group, 45 men with CP/CPPS with no treatment, at the beginning and at the end of the study.

We assessed the QoL according to NIH-CPSI (Figure 4). In the control group, the mean QoL decreased slightly from 8.47 to 8.33, whereas in the treatment group the mean QoL decreased from 8.11 to 2.98. For the control group, the z value was −0.420 at the significance level of 0.675 with a *p* value > 0.05. For the treatment group, the z value was −5.661 at the significance level *p* value < 0.001. These results indicate that the treatment with the therapeutic device decreased the QoL score significantly, while in the control group it decreased slightly, see Figure 4.

## 4. Discussion

Examined before and after a 6-month treatment period with the therapeutic device, the patients with BPH reported a significant improvement to the disturbing LUTS and QoL. Moreover, after the use of the therapeutic device the PV level decreased and uroflowmetry Q_max_ significantly increased. Changes in NIH-CPSI-QoL scores have shown the positive results in improving QoL. More details on the influence of thermobalancing therapy on men with CP will be discussed in another article. At the same time, in the no-treatment groups the symptoms and parameters worsened.

These obtained data indicate that thermobalancing therapy is effective for BPH and CP. The observational clinically controlled study was used on both separate trials for CP and BPH. The studies were not randomized, which may limit the accuracy of the results. Of course, having “placebo” or “sham” group as controls could provide more confidence in the outcomes. However, most men with CP and pelvic pain have serious mental problems, so some experts believe that psychological problems are part of the cause of CP/CPPS [14]. Therefore, it was difficult to suggest to men with CP/CPPS that they wear something useless for six months. Usually, patients with CP/CPPS felt healthier within weeks; therefore, they used the device as was required. We had similar thoughts about patients with an enlarged prostate, as the health-related QoL of patients with BPH is considered poor, and their psychological well-being severely affected. Postvoid residual urine, lower urinary tract symptoms, anxiety, and depression are identified as being significant predictive factors of the health-related QoL of patients with an enlarged prostate [15].

LUTS associated with BPH is a highly impactful condition that is often undertreated. LUTS/BPH have a major impact on men, their families, health services, and society. Men with LUTS secondary to BPH should not simply accept their symptoms as part of the ageing process, but should be encouraged to consult their physicians if they have bothersome symptoms [16]. In the last decade, the opinion of the necessity of medical/surgical treatment of BPH has been challenged. BPH/LUTS should not be viewed as an inevitable disease of older people but as part of the aging process which can be prevented [17]. Moreover, research that has included a total of 2,620,269 patients with BPH who were treated within 5 years has shown that medical treatment was interrupted for approximately 16% of patients. Therefore, it is necessary to improve the level of care for men with BPH [18].

As thermobalancing therapy is free from side effects, it can play an important role in the prevention of BPH development and progression. Thermobalancing therapy is entirely different from common heating treatments because it regulates the affected organ’s temperature locally, maintaining it within the normal body temperature range. All other thermotherapies can damage organs, because the high temperatures injure normal cells. On the other hand, low temperatures decrease cellular metabolism and, as a result, interfere with natural replenishment and healing.

The therapeutic device tightly applies the thermoelement to the skin in the projection of the prostate overcoming the skin barrier spreading energy inside the body. Allen hypothesized that this therapy long-term effect on the prostate improves blood circulation at the capillary level. This view is supported by various data; for instance, in the last decade, the pathogenesis of BPH began to be considered from the perspective of vascular dysfunction [19], chronic ischemic tissue [20], and increasing the pressure in the prostate [21].

However, other investigators state that, apart from the old concept of mainly hormone-dependent prostate growth, new ideas about the cause of BPH include an inflammatory factor [22]. Therefore, materials presented at a satellite symposium entitled, “Inflammation and Prostatic Diseases: From Bench to Bedside,” held during the 2015 annual meeting of the European Association of Urology in Madrid, Spain, revealed that reducing chronic inflammation is a target for the treatment of BPH [23].

NSAIDs are commonly used as anti-inflammatory drugs worldwide, but these medications are associated with side effects in the gastrointestinal (GI) tract. Furthermore, these drugs can damage gastric and duodenal mucosa, and even the esophagus [24]. Certain NSAIDs can increase the risk of heart attack and stroke, especially in long-term use; therefore, prescription of this medication should be avoided in patients at high risk of cardiovascular disease [25].

The therapeutic device, in contrast with NSAIDs and long-term BPH medications, provides side-effect-free treatment that targets the pathological nidus in the prostate continuously for a prolonged period of time, *i.e.*, for days, months, or even years, maintaining the accumulated temperature. Allen’s research on the origin of diseases suggests the causal root of chronic internal diseases, including prostate enlargement, namely capillary expansion.

This conclusion is based on two functional physiological properties of capillaries which are activated by an irritating factor, *i.e.*, a trigger. The constriction of capillaries in response to an irritating trigger develops local micro-hypothermia. It is this focus on hypothermia, which in turn becomes a constant irritant maintaining illness, making a disease chronic. In response to irritation (*i.e.*, a trigger-initiator and later focus on hypothermia) and in order to eliminate them, the blood flow increases through the spontaneous expansion of the capillary net locally. The formation of new capillaries is essentially the growth of the excess tissue that leads to an increased pressure inside the prostate and its enlargement.

For the purpose of a targeted treatment for the secondary focus on hypothermia inside the organ, Allen suggested thermobalancing therapy enabled by a therapeutic device. This device contains a natural thermoelement which accumulates body heat, becoming a source of energy itself. There is no battery or electricity involved. Moreover, it should be noted that treatments with imposed heat over 40 °C (104 Fahrenheit) can be damaging, because the high temperatures destroy delicate cells of the living organism. We believe that the use of the therapeutic device by keeping the right temperature in the projection of the prostate gland improves blood circulation at the capillary level, stopping and reversing prostate swelling and enlargement, and easing the BPH and CP symptoms.

## 5. Conclusions

Both clinical trials of CP and BPH highlight that the therapeutic device is potentially able to target pathogenetic component in the prostate gland. Thermobalancing therapy is a new prospect for safe and effective physiotherapeutic intervention in BPH treatment. The reduction of the PV after use of the therapeutic device, termed Allen’s Device, correlates with a significant decrease in urinary symptoms and an improvement of QoL in patients with an enlarged prostate and CP.
